# Systematic Review and Meta-Analysis of Inflammatory Biomarkers in Individuals Exposed to Radon

**DOI:** 10.3390/biomedicines13020499

**Published:** 2025-02-17

**Authors:** Anel Lesbek, Yasutaka Omori, Meirat Bakhtin, Polat Kazymbet, Shinji Tokonami, Nursulu Altaeva, Danara Ibrayeva, Yerlan Kashkinbayev

**Affiliations:** 1 Institute of Radiobiology and Radiation Protection NJSC, Astana Medical University, Astana 010000, Kazakhstan; omar.a@amu.kz (A.L.); bakhtin.m@amu.kz (M.B.); kazimbet.p@amu.kz (P.K.); danaraibrayeva@gmail.com (D.I.); 2 Institute of Radiation Emergency Medicine, Hirosaki University, 66-1 Hon-cho, Hirosaki 036-8564, Aomori, Japan; ys-omori@hirosaki-u.ac.jp (Y.O.); tokonami@hirosaki-u.ac.jp (S.T.); 3 Department of Medical Genetics and Molecular Biology NJSC, Astana Medical University, Astana 010000, Kazakhstan; altaeva.n@amu.kz

**Keywords:** radon exposure, inflammatory biomarkers, biological fluids, radon-induced health effects, systematic review, meta-analysis, residential radon exposure, occupational radon exposure, C-reactive protein, tumor necrosis factor-alpha (TNF-α)

## Abstract

**Background/Objectives**: Radon is a significant carcinogen, particularly as a leading cause of lung cancer among non-smokers. While its carcinogenic effects are well documented, the relationship between radon exposure and inflammatory reactions remains underexplored. This systematic review investigates inflammatory biomarkers in individuals exposed to chronic radon exposure and conducts a meta-analysis on serum C-reactive protein (CRP) and tumor necrosis factor-alpha (TNF-α) levels. **Methods**: A systematic search was conducted in PubMed, Scopus, Web of Science, ScienceDirect, and Google Scholar using the keywords “radon” AND “inflammation biomarkers” following established guidelines. Studies reporting inflammatory biomarker levels in biological fluids of human participants exposed to residential or occupational radon were included. Statistical analyses, including pooled mean estimates, influence analysis, publication bias, and meta-regression, were performed in RStudio. **Results**: Ten studies involving 33,099 individuals met the inclusion criteria. Eight studies focused on residential radon exposure, and two examined occupational exposure among uranium miners. Inflammatory biomarkers were analyzed in serum, bronchoalveolar lavage fluid, and saliva. Among individuals exposed to high residential radon levels, serum CRP and TNF-α were the most frequently assessed biomarkers, with pooled mean levels of 2.11 mg/L (95% CI, 1.32–2.89) and 2.20 pg/mL (95% CI, 0.25–4.64), respectively. **Conclusions**: Serum CRP and TNF-α levels appear lower in adults with chronic radon exposure, suggesting potential anti-inflammatory effects despite radon’s established carcinogenicity. Future longitudinal studies using standardized methods are crucial to elucidate the long-term health impacts of radon exposure.

## 1. Introduction

Radon (Rn-222) is an odorless, tasteless, and radioactive gas that is a byproduct of the nuclear decay of radium (Ra-226). Radon is the primary source of natural radiation exposure to humans. It is formed within the Earth’s crust as part of the uranium decay series and can seep into the atmosphere, water, and soil [1]. Radon concentrations differ depending on environmental and structural factors. Elevated levels of radon are commonly found in enclosed spaces such as caves, mines, and buildings due to its ability to accumulate in poorly ventilated areas [2]. Radon levels inside buildings are often elevated due to soil infiltration and pressure differences between indoor and outdoor environments [2]. This paper highlights the two main pathways of radon exposure: residential and occupational.

The presence of radon in a residential or an occupational setup is dependent upon the underlying geological environment and building type, as well as the lifestyle being led [3]. High radon levels have been recorded in areas with soil rich in uranium, homes with poor ventilation, and workplaces such as uranium mines [4]. Uranium mining activities disturb the natural equilibrium of radioactive elements in the Earth’s crust, releasing radon gas into the surrounding environment [4]. Workers in these settings face chronic exposure to high radon levels, often exceeding those encountered in residential environments. The restricted spaces and poor ventilation in mines lead to higher radon levels, which significantly heightens the health risks for miners. This occupational exposure has historically been associated with an increased risk of lung cancer [5]. While substantial research has illuminated radon’s carcinogenic properties, particularly its role in lung cancer etiology, systematic investigations into its broader biological effects, especially its relationship with inflammatory biomarkers, remain sparse. This gap in the environmental health literature highlights the need for focused research to assess the non-carcinogenic pathways through which radon affects human health.

It is well documented that radon is a significant carcinogen, especially as a major cause of lung cancer in non-smokers. According to the World Health Organization, radon exposure accounts for approximately 3% to 14% of global lung cancer cases, with the variation largely dependent on regional radon levels and smoking prevalence [6]. Interestingly, despite its known hazards, radon has historically been used in balneotherapy. In this context, low-dose radon inhalation and water therapies have reportedly exhibited pain-relieving and anti-inflammatory properties [7].

Inflammation, a cornerstone of the body’s response to environmental insults, is a key mechanism linking radon exposure to broader health outcomes. Biomarkers such as C-reactive protein (CRP) and tumor necrosis factor-alpha (TNF-α) are central to the inflammatory cascade and serve as indicators of systemic inflammatory status [8,9]. These markers are not only pivotal in understanding inflammation’s role in disease progression but also valuable in assessing the impact of environmental exposures on human health. Multiple studies suggest that radon’s ionizing radiation may influence inflammatory pathways, contributing to pain reduction in clinical experience [10].

Although radon exposure and its health effects are often discussed, there is a notable absence of comprehensive reviews examining its association with inflammatory reactions. This systematic review seeks to bridge this gap by investigating inflammatory biomarkers across various biological fluids in individuals exposed to chronic levels of radon exposure. Furthermore, as a secondary aim, we conduct a meta-analysis of the most frequently analyzed inflammatory biomarkers based on our systematic review findings, specifically mean serum CRP and TNF-α levels, among participants with chronic residential radon (CRR) exposure. This dual approach not only bridges critical gaps in the literature but also offers insights to inform public health strategies and future research directions.

## 2. Materials and Methods

The study protocol has been registered with PROSPERO, the International Prospective Register of Systematic Reviews maintained by the National Institute for Health Research [11] (ID: CRD42025630827).

### 2.1. Search Strategy

An initial search of the PROSPERO database to identify registrations of comparable studies revealed no similar systematic review protocols. Subsequently, a systematic search was conducted across five literature databases: PubMed, Scopus, Web of Science, ScienceDirect, and Google Scholar. The search began on 21 November 2024 and concluded on 20 December 2024. The search strategy employed the following keywords: “radon” OR “radioactive gas” AND “inflammation biomarkers” OR “inflammatory markers”. No restrictions were placed on publication date; however, the results were limited to English-language publications and studies conducted on humans. Filters were applied, where available, to include only research articles and exclude other types of publications

### 2.2. Eligibility Criteria

The eligibility criteria for study inclusion were as follows: studies involving human participants, including both adults and children, with documented radon exposure in occupational or residential settings. Eligible studies were required to quantitatively measure radon exposure levels or provide background data on radon levels and assess inflammatory biomarkers in biological fluids such as serum, urine, alveolar fluid, or saliva. Original research articles presenting primary data, including cohort studies with cross-sectional data on inflammatory biomarkers, case–control studies, and cross-sectional studies published in peer-reviewed journals, were included. Studies were excluded if they focused on animal models or in vitro research; examined the efficacy of radon therapy in any form; or were published as reviews, conference abstracts, commentaries, editorials, or short communications. Additional exclusions applied to studies lacking information on inflammatory biomarkers and publications not available in English.

### 2.3. Selection of Studies and Data Extraction

The literature review and synthesis were carried out in adherence to the guidelines outlined by the Preferred Reporting Items for Systematic Reviews and Meta-Analyses (PRISMA) [12]. Two authors (AL and YK) dependently screened the titles and abstracts of the search results, following the removal of duplicates, to assess their relevance. Full-text articles of studies that met the initial screening criteria were retrieved and evaluated against the pre-specified inclusion and exclusion criteria. Data extracted from the eligible studies included the first author’s last name, year of publication, study design, region and country, population description, sample size, age, type of radon exposure (residential or occupational), radon level assessment method, radon exposure level, biomarkers assessed (pro-inflammatory, anti-inflammatory, and others), and biomarker levels. Two authors independently performed the data extraction process. Any discrepancies were resolved through consultation with a third author (YO) to ensure consensus among all three authors involved in study selection and data extraction.

### 2.4. Risk of Bias (Quality) Assessment

The studies included in this review were assessed for risk of bias (quality) using the Newcastle–Ottawa Scale (NOS) for case–control studies and an adapted version for cross-sectional studies, as recommended by the Cochrane Non-Randomized Studies Methods Working Group [13]. The NOS for case–control studies evaluates each study based on eight criteria, which are divided into three main categories: Selection of study groups (four items), Comparability of these groups (one item), and Exposure (three items). Each criterion can receive up to one point, while the comparability criterion can be awarded a maximum of two points. The total score ranges from 0 to 9, with higher scores indicating better study quality.

For cross-sectional studies, the adapted NOS assesses each study across six criteria, divided into three main categories: Selection (three items), Comparability (one item), and Outcome (two items). Each criterion can receive up to one point, and the comparability criterion can be awarded a maximum of two points, resulting in a total score ranging from 0 to 7, with higher scores reflecting better study quality.

Risk of bias (quality) assessments were independently conducted by two authors (AL and YK) after they had agreed upon the assessment procedures. Inter-rater agreement between the two assessors was calculated by a third author (YO). In this review, studies scoring seven points or higher for case–control studies and five points or higher for cross-sectional studies were classified as having satisfactory quality and included in the systematic review [14,15].

### 2.5. Qualitative Data Synthesis

The data synthesis involved organizing the included studies to ensure a clear and systematic presentation of the findings. Studies were first sorted chronologically by publication year and then alphabetically by the last name of the first author. To facilitate a structured comparison, the studies were categorized based on the types of biological fluids assessed in participants, with the results presented in a table. For inflammation biomarkers, biomarker levels were reported separately for exposed and non-exposed groups, where such data were available, to highlight differences associated with radon exposure. Biomarkers were divided into pro-inflammatory (e.g., CRP, TNF-α, ICAM-1, IL-1β, IL-2, INF-γ, IL-6, IL-8, MPO, 8-epi-PGF2α, VCAM-1, MCP-1, P-selectin, TNFR-2, MIF, and VEGF), anti-inflammatory (e.g., IL-4 and IL-10), and other biomarkers.

### 2.6. Meta-Analysis

The pooled mean serum CRP and TNF-α levels for participants with high residential radon exposure were calculated using a random-effects model for meta-analysis in RStudio (version 4.3.2), employing the “meta” and “metafor” packages. The results from the random-effects model were visualized through forest plots. Heterogeneity among the studies was evaluated using the I^2^ statistic, accompanied by an influence analysis. Publication bias was assessed using Egger’s test, with results illustrated through funnel plots. A meta-regression analysis was conducted to examine the effect of age on the pooled mean serum CRP and TNF-α levels. A subgroup analysis was not performed due to the limited number of studies included in the meta-analysis.

### 2.7. Evaluation of Certainty of Evidence

We evaluated the certainty of evidence using the Grading of Recommendations Assessment, Development, and Evaluation (GRADE) framework, following the guidelines provided in the Cochrane Handbook for Systematic Reviews of Interventions [16]. The evaluation encompassed five domains: Risk of Bias, assessed via the NOS checklist described above; Inconsistency, gauged by the I^2^ statistic, with I^2^ > 75% labeled as “Serious”, I^2^ > 50% as “Moderate”, and I^2^ > 25% as “Not serious”; Indirectness, examined based on PICO criteria; Imprecision, determined by whether the 95% confidence interval (CI) of the pooled estimate crossed the threshold of interest; and Publication Bias, evaluated using funnel plots and Egger’s test. This assessment adhered to the procedures detailed in the research notes on GRADE evaluation in systematic reviews [17]. Certainty of evidence was calculated in RStudio, adhering to those guidelines and using the “grade” package.

## 3. Results

### 3.1. Included Study Characteristics

**Figure 1:**
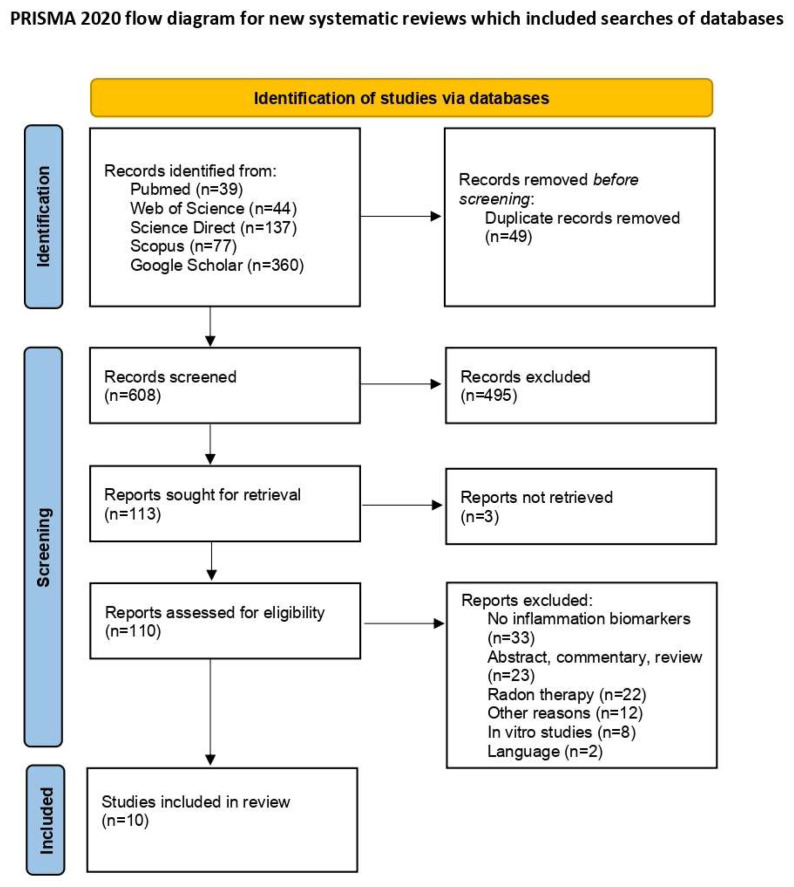
PRISMA flow diagram of study selection process [12].

The initial database search identified 657 articles. After removing 49 duplicates, 608 unique articles remained for screening. Of these, 110 articles were selected for full-text review following the exclusion of 495 irrelevant titles and three articles for which full texts were unavailable. Upon further assessment, 10 articles met the inclusion criteria. The exclusions included 33 studies that lacked information on inflammatory biomarkers; 23 studies that were abstracts, commentaries, or reviews; 22 studies that focused on radon therapy rather than residential or occupational radon exposure; 12 studies excluded for other reasons; 8 in vitro studies; and 2 studies not published in English [18,19]. A PRISMA flow diagram detailing the study selection process is presented in Figure 1 [12].

The included studies were published between 2000 and 2023. Five studies were conducted in the United States; two in Iran; and one study each in Germany, Thailand, and Indonesia. Eight studies focused on residential radon exposure, including one study that exclusively assessed children, while the others focused on adults. Two studies examined occupational radon exposure among uranium miners, one conducted in Germany and the other in the United States. Three studies included participants diagnosed with cancer. In total, 33,099 participants were assessed. Radon level assessments and the reported radon levels varied across the included studies. Eight studies assessed inflammation biomarker levels in serum, with one study assessing both serum and human bronchial epithelial cells. One study each assessed the bronchoalveolar lavage fluid and saliva. Detailed information on the included articles is provided in Table 1.

### 3.2. Inflammation Biomarker Levels in Various Biological Fluids in Studies on Radon Exposure

Table 2 provides a summary of data on inflammation biomarker levels across various biological fluids, including serum, bronchoalveolar lavage fluid, and saliva. Six out of ten studies presented data on inflammation biomarker levels among radon-exposed and non-exposed participants. Of these, one study assessed biomarkers in bronchoalveolar lavage fluid, while the remaining studies evaluated them in serum. Among participants with CRR exposure, the most frequently assessed pro-inflammatory biomarkers in serum were CRP, reported in three studies and eight participant groups, and TNF-α, reported in three studies and four groups. These were followed by ICAM-1, assessed in two studies with three participant groups, IL-2 in two studies, INF-γ in two studies, and IL-6 in two studies. Other pro-inflammatory biomarkers assessed in serum included MPO in one study, 8-epi-PGF2α in one study, VCAM-1 in one study, MCP-1 in one study, P-selectin in one study, TNFR-2 in one study, MIF in one study, and VEGF in one study. Among the anti-inflammatory biomarkers in serum, IL-4 was assessed in three studies, while IL-10 was assessed in two studies. In bronchoalveolar lavage fluid, TNF-α was the only pro-inflammatory biomarker evaluated. In saliva, the assessed pro-inflammatory biomarkers included CRP, IL-1β, IL-6, IL-8, and TNF-α. Notably, no anti-inflammatory biomarkers were assessed in either bronchoalveolar lavage fluid or saliva.

### 3.3. Risk of Bias (Quality) Assessment

All the case–control studies included had a NOS score of 7 or higher out of 8, while all cross-sectional studies had a NOS score of 6 out of 7. These scores suggest that the studies were of high quality and had a low risk of bias, as shown in Table 3.

### 3.4. Meta-Analysis of Mean Serum CRP and TNF-α Levels Among Participants with Chronic Residential Radon Exposure

**Figure 2:**
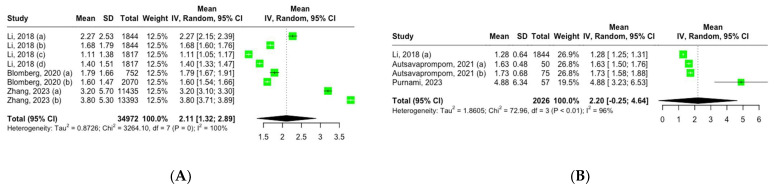
Forest plot of mean inflammation biomarker levels among participants with chronic residential radon exposure: (**A**) CRP levels; (**B**) TNF-α levels. Abbreviations: SD—standard deviation. Group definitions: Li, 2018 (a) [26] —offspring cohort cycle 7; Li, 2018 (b) [26] —offspring cohort cycle 8; Li, 2018 (c) [26]—third-generation cohort cycle 1; Li, 2018 (d) [26]—third-generation cohort cycle 2; Blomberg, 2020 (a) [27] —first visit; Blomberg, 2020 (b) [27] —all visits; Zhang, 2023 (a) [30] —never smokers; Zhang, 2023 (b) [30] —ever smokers; Autsavapromporn, 2021 (a) [28] —high-residential-radon-exposure participants; Autsavapromporn, 2021 (b) [28] —high-residential-radon-exposure participants with lung cancer.

Three studies, encompassing eight groups, reported the mean serum CRP levels among adult participants with CRR exposure. Using a random-effects model, the pooled mean CRP level across these groups was 2.11 mg/L (95% CI, 1.32–2.89), showing high heterogeneity: I^2^ = 100%, Q (df = 7) = 3264, *p* = 0 (Figure 2A).

Three studies comprising four groups reported the mean serum TNF-α levels among adult participants with CRR exposure. Using a random-effects model, the pooled mean TNF-α level across these groups was 2.20 pg/mL (95% CI, 0.25–4.64), showing high heterogeneity: I^2^ = 96%, Q (df = 3) = 72.96, *p* < 0.01 (Figure 2B).

**Figure 3:**
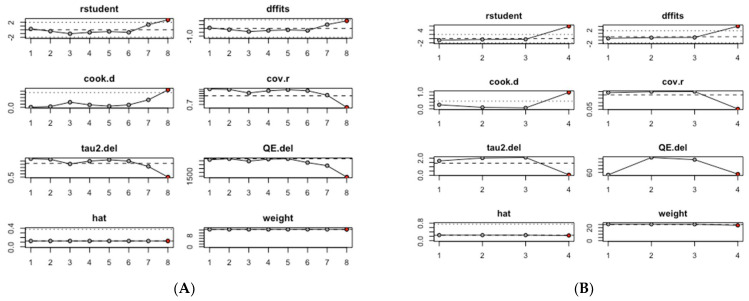
Influence analysis of mean inflammation biomarker levels among participants with chronic residential radon exposure: (**A**) CRP levels; (**B**) TNF-α levels.

An influence analysis was conducted to identify which studies had the greatest impact on the pooled estimates of mean serum CRP and TNF-α levels. Among the studies assessing serum CRP levels, the pooled mean CRP value was predominantly influenced by study #8, Zhang, 2023 (b) [30], which focused on ever smokers (Figure 3A). Similarly, in the group of studies evaluating serum TNF-α levels, the pooled mean TNF-α value was primarily influenced by study #4, Purnami, 2023 [29] (Figure 3B).

**Figure 4:**
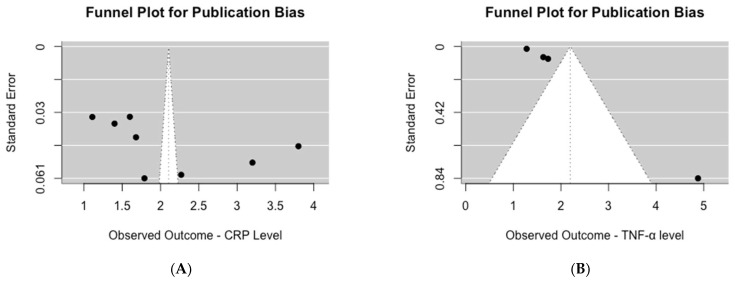
Publication bias assessment of mean inflammation biomarker levels among participants with chronic residential radon exposure: (**A**) CRP levels; (**B**) TNF-α levels.

Upon visual inspection of the funnel plot for studies assessing serum CRP levels, asymmetry was evident (Figure 4A). This observation was further supported by the significant results of Egger’s test for publication bias (*p* = 0.04). Similarly, for studies evaluating serum TNF-α levels, visual inspection of the funnel plot also revealed asymmetry (Figure 4B), which was confirmed by the highly significant results of Egger’s test for publication bias (*p* < 0.0001).

**Figure 5:**
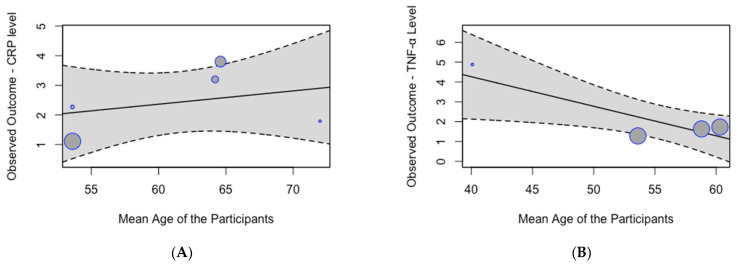
Meta-regression analysis of mean inflammation biomarker levels among participants with chronic residential radon exposure by age: (**A**) CRP levels; (**B**) TNF-α levels.

A meta-regression analysis did not identify a significant effect of age on the pooled mean serum CRP levels (*p* = 0.63; Figure 5A). However, the analysis demonstrated a significant effect of age on the pooled mean serum TNF-α levels (*p* = 0.01; Figure 5B), indicating that mean serum TNF-α levels decrease with age.

### 3.5. Evaluation of Certainty of Evidence

The findings from the GRADE certainty assessment, as detailed in Table 4, indicate that the pooled mean levels of CRP and TNF-α exhibit a low degree of certainty. Consequently, these results should be interpreted with caution.

## 4. Discussion

Our systematic review aimed to investigate the inflammatory biomarkers present in various biological fluids among individuals exposed to chronic levels of radon and to conduct a meta-analysis of the mean serum CRP and TNF-α levels in participants with CRR exposure. Inflammatory biomarkers were assessed in serum, bronchoalveolar lavage fluid, and saliva. Among participants with CRR exposure, the most frequently evaluated pro-inflammatory biomarkers in serum were CRP and TNF-α, followed by ICAM-1, IL-2, INF-γ, IL-6, MPO, 8-epi-PGF2α, VCAM-1, MCP-1, P-selectin, TNFR-2, MIF, and VEGF. For anti-inflammatory biomarkers in serum, IL-4 and IL-10 were analyzed. In bronchoalveolar lavage fluid, TNF-α was assessed, while in saliva, the evaluated pro-inflammatory biomarkers included CRP, IL-1β, IL-6, IL-8, and TNF-α. No anti-inflammatory biomarkers were assessed in either bronchoalveolar lavage fluid or saliva.

This systematic review and meta-analysis also revealed an association between CRR exposure and low levels of inflammatory biomarkers, particularly CRP and TNF-α. The pooled mean serum CRP level among participants with CRR exposure was 2.11 mg/L (95% CI, 1.32–2.89), while the pooled mean serum TNF-α level was 2.20 pg/mL (95% CI, 0.25–4.64). Based on the low certainty of evidence using the GRADE framework, the findings of this study should be interpreted with caution. Radon is a well-established carcinogen known to induce oxidative stress and DNA damage. However, our findings suggest that radon exposure may be associated with reduced systemic inflammation, although the mechanisms underlying this observation remain unclear. Importantly, these results should not be interpreted as evidence of a direct anti-inflammatory effect of radon but, rather, as an indication of a complex biological response that warrants further investigation.

To better understand the potential impact of radon on lung-specific inflammation, we propose a hypothetical scheme linking radon exposure to changes in CRP and TNF-α levels in the lung. Radon, as an alpha particle emitter, induces oxidative stress and DNA damage in lung tissue, which may trigger a cascade of cellular responses [34]. Initially, radon-induced DNA damage activates repair mechanisms and inflammatory pathways, leading to the release of pro-inflammatory cytokines such as TNF-α [35]. However, with chronic exposure, the sustained oxidative stress and DNA damage may lead to immune modulation, characterized by a downregulation of inflammatory responses as a protective mechanism [36]. This could explain the observed reductions in serum CRP and TNF-α levels in individuals with CRR exposure. Additionally, radon exposure may alter the lung microenvironment, affecting the production and clearance of these biomarkers. For instance, radon-induced epithelial damage could impair the local production of TNF-α, while systemic immune suppression might reduce CRP synthesis in the liver [37]. This proposed scheme highlights the need for future studies to investigate the temporal dynamics of biomarker changes and to explore the role of lung-specific mechanisms in mediating the observed effects.

Our findings suggest a complex link between radon exposure and its impact on human health. While chronic radon exposure appears to reduce levels of certain inflammatory biomarkers, potentially indicating anti-inflammatory effects, it remains strongly linked to an elevated risk of lung cancer. The health risks of radon are mainly due to its ionizing radiation, which causes DNA damage and triggers carcinogenic processes [6]. The International Commission on Radiological Protection (ICRP) highlights that even minimal exposure to radon carries a lung cancer risk, underscoring its critical public health importance and recommending an integrated strategy for controlling radon exposure [38]. A recent meta-analysis of fifty-five studies confirms that CRR exposure is associated with elevated incidence of lung cancer and pediatric leukemia [39]. The paradoxical association can be partially explained by the dual biological effects of radon’s ionizing radiation, which may suppress systemic inflammation while concurrently inducing DNA damage, chromosomal abnormalities, and alterations in the cell cycle [34]. Furthermore, low levels of inflammatory biomarkers such as CRP and TNF-α observed in radon-exposed individuals may reflect an adaptive response or immune suppression, which could inadvertently compromise the body’s ability to detect and eliminate cancerous cells [10]. The observed reduction in inflammatory biomarkers could also explain the therapeutic application of radon in the form of hot spas for patients with degenerative and inflammatory conditions accompanied by pain [10]. However, in scenarios involving non-chronic exposure relevant to radon therapy for chronic inflammatory diseases, there is a complete lack of epidemiological data to estimate the associated cancer risk [10].

The findings of this study have important practical implications. Future research should prioritize longitudinal studies designed to evaluate the potential benefits and risks of radon therapy for chronic inflammatory diseases. These studies should systematically measure the effects of controlled radon exposure on inflammatory biomarkers while simultaneously monitoring long-term cancer incidence rates. Additionally, experimental studies could elucidate the underlying biological mechanisms by which low-dose, short-term radon exposure exerts anti-inflammatory effects, potentially through pathways involving oxidative stress modulation or immune response alteration. To ensure robust and generalizable findings, future investigations should focus on diverse populations, including individuals with pre-existing inflammatory conditions, varying genetic predispositions, and different environmental radon exposure baselines.

This study has several limitations. First, the included studies exhibited significant heterogeneity in radon exposure assessment methods, biomarker measurement techniques, and participant demographics. Consequently, we were unable to compare high- and low-radon-exposure groups effectively. Second, the small number of studies included in the meta-analysis limits the generalizability of the findings. Third, publication bias, as indicated by Egger’s test, suggests that studies reporting null or negative findings may be underrepresented. Fourth, the cross-sectional nature of most included studies precludes causal inferences. Lastly, this study does not account for confounding factors that could influence inflammatory biomarker levels independently of radon exposure, such as smoking status, occupational exposure of residential participants, pre-existing medical conditions, and dietary habits, among others. These confounders may introduce variability into the observed relationships between radon exposure and inflammatory biomarkers, potentially limiting the interpretability of the findings. The included articles lacked uniform and comprehensive information on the aforementioned confounders, which precluded our ability to systematically collect and analyze these variables in the context of the present analysis.

## 5. Conclusions

In summary, this systematic review emphasizes that CRP and TNF-α are the most commonly studied pro-inflammatory biomarkers in serum among individuals exposed to high levels of residential or occupational radon. The meta-analysis findings reveal that these biomarkers are lower in adults with CRR exposure, pointing to potential anti-inflammatory effects, even as radon remains a well-recognized carcinogen. These insights highlight the complex health implications of radon exposure, extending beyond its established link to cancer. Moving forward, it is essential for future research to focus on longitudinal studies using standardized methods to better understand the long-term health impacts of short-term radon exposure used for health benefits. By addressing these research gaps, we can deepen our understanding of radon’s diverse health effects and create a stronger foundation for informed public health strategies.

## Figures and Tables

**Table 1:** Description of the included studies.

First Author, Year	Study Design	Region/Country	Population Explored	Sample Size	Age Mean ± SD or Median (25–75%) ^1^	Residential/ Occupational	Radon Level Assessment	Radon Level
**Serum biomarkers**								
Attar, 2007 [20]	Case–control	Taleshmahaleh or Chaparsar village—exposed group and Sephydtameshk or Daryaposhteh village—not exposed group, Iran	Residents	100	20 and 50 years of age	Residential	High background radiation and low background radiation [21,22,23]	Background ionizing radiation level: 260 mSv/year. Mean indoor ^222^Rn levels: 2255 Bq/m^3^ in autumn and 3235 Bq/m^3^ in winter. [21,22,23]
Molaie, 2012 [24]	Case–control	Taleshmahaleh or Chaparsar village—exposed group and Sephydtameshk or Daryaposhteh village—not exposed group, Iran	Residents of the villages	102	HRR: 37 (31–44) LRR: 32 (23–50)	Residential	High background radiation and low background radiation [21,22,23]	Background ionizing radiation level: 260 mSv/year. Mean indoor ^222^Rn levels: 2255 Bq/m^3^ in autumn and 3235 Bq/m^3^ in winter. [21,22,23]
Leng, 2015 [25]	Case–control	Saccomanno Uranium Miner cohort, United States	Former uranium miners	Squamous cell carcinoma: 242 Control: 336	Squamous cell carcinoma age at death: 62.6 ± 10.6 Control age at death: 69.2 ± 12.5	Occupational	Cumulative radon exposure at work, expressed as WLM, was evaluated	0.8 WLM for cancer population and 0.7 WLM for controls
Li, 2018 [26]	Cross-sectional data of the cohort study	In a radius extending 50 km from the Harvard Supersite air pollution monitoring station, Boston, MA, United States	Framingham Offspring cohort and the first and second examinations of the third-generation cohort	4056 participants or 6963 observations	Overall cohort: 53.6 ± 14.2	Residential	The gross beta activity data was sourced from the RadNet monitoring network of the U.S. EPA (decay of short-lived radon progenies (e.g., 214Pb, 214Bi))	^1^ 1-day moving average: 0.276 ± 0.084 Bq/m^3^ Gross beta radioactivity (offspring cohort): 0.295 ± 0.083 Bq/m^3^ Gross beta radioactivity (third-generation cohort): 0.305 ± 0.091 Bq/m^3^
Blomberg, 2020 [27]	Cross-sectional data of the cohort study	Boston, MA; Concord, NH; Hart- ford, CT; Providence, RI; and Worcester MA, United States	NAS participants	752 participants and 2070 total visits	First visit: 72 ± 7, all visits: 75 ± 7	Residential	Gross beta radiation levels were monitored under the EPA’s RadNet initiative (decay of short-lived radon progenies (e.g., 214Pb, 214Bi))	Particle radioactivity (4–10 Bq/m^3^): first visit: 2.7 ± 1.1 Bq/m^3^; all visits: 2.6 ± 1.1 Bq/m^3^
Autsavapromporn, 2021 [28]	Case–control	The Hang Dong, Muang, Saraphi and San Pha Tong districts of Chiang Mai city, Thailand	Residents	150: 75 participants with lung cancer, 75 participants without lung cancer, 75–LRR, 50—HRR group	Lung cancer: 60.3 ± 10.8; LRR: 61.2 ± 7.1; HRR: 58.8 ± 8.8	Residential	To measure the radon activity concentration, a RADUET passive monitor employing a solid-state track detector (CR-39) was utilized, which effectively discriminates between radon and thoron.	The study area classifies radon levels into three distinct categories: “low” (<44 Bq/m^3^), “moderate” (44–70 Bq/m^3^), and “high” groups (>70 Bq/m^3^) based on indoor radon concentration in the dwellings
Purnami, 2023 [29]	Case–control	Tande-Tande—a sub-village in Northern Botteng village, Mamuju regency in Simboro sub-district, Indonesia	Residents	HRR: 57; LRR: 53	HRR: 40.09 ± 15.19. LRR: 42.28 ± 11.94	Residential	To measure the radon activity concentration, a RADUET passive monitor employing a solid-state track detector (CR-39) was utilized, which effectively discriminates between radon and thoron.	The mean indoor radon-222 concentration in the area reaches 1644 Bq/m^3^
Zhang, 2023 [30]	Cross-sectional data of the cohort study	Blacks and residents of the Southeastern states such as Arkansas, Alabama, Georgia, Louisiana, Mississippi, North Carolina, South Carolina, and Tennessee were oversampled, continental United States	REGARDS study participants: never smokers Zone 3 (30.34 Bq/m^3^); never smokers Zone 1 + Zone 2 (106.19 Bq/m^3^) ever smokers Zone 3 (30.34 Bq/m^3^); ever smokers Zone 1 + Zone 2 (106.19 Bq/m^3^)	Never smokers: 12 453 Ever smokers: 14 495	Never smokers Zone 3—64.2 ± 9.8; never smokers Zone 1 + Zone 2—64.8 ± 9.9; ever smokers Zone 3 —64.6 ± 9.0; ever smokers Zone 1 + Zone 2—64.9 ± 8.8	Residential	(1) State/EPA residential radon survey (2) County-level radon measures from Lawrence Berkeley National Laboratory	^1^ The mean radon concentration reached 30.34 Bq/m^3^ in Zone 3 and 106.19 Bq/m^3^ In Zone 1/2
**Bronchoalveolar lavage fluid**								
Popp, 2000 [31]	Case–control	The Wismut company miners, Germany	Former uranium miners	Exposed URAN: 106. Not exposed: CON: 23; FIBR: 16; CANC: 90	URAN: 67 ± 5; CON: 60 ± 7; FIBR: 60 ± 12; CANC: 63 ± 9	Occupational	German Wismut uranium miner’s cohort Radiation Protection dosimetry study [32]	Up to 1955, radon expo-sure was in the range of 30–300 WLM per year and dust exposure in the range of 10–100 mg/m^3^
**Saliva**								
Taylor, 2022 [33]	Cross-sectional	Eastern Nebraska and Western Iowa, United States	Children aged 6 to 14 years	68	10.27 ± 2.59	Residential	Short-term home radon testing kit	^1^ Mean radon concentration: 244.26 ± 274.91 Bq/m^3^

^1^—units were recalculated to Bq/m^3^. Abbreviations: CANC—patients with lung cancer; CON—healthy controls; EPA—Environmental Protection Agency; FIBR—patients with lung asbestosis or fibrosis of the lung; HRR—high residential radon; LRR—low residential radon; NAS—Normative Aging Study; URAN—uranium miners; WLM—working level month.

**Table 2:** Summary of inflammation biomarker levels in various biological fluids.

First Author, Year Participants	Biomarkers in Exposed: (Mean ± SD) or (Median (25–75 Percentile))	Biomarkers in Non-Exposed: (Mean ± SD) or (Median (25–75 Percentile))
**Serum biomarkers**		
Attar, 2007 [20] **Participants:** High background radiation vs. low background residential radiation	**Pro-Inflammatory:** IL-2 (pg/mL): 399 ± 221 INF-γ (OD): 0.12 ± 0.04 **Anti-Inflammatory:** IL-4 (pg/mL): 220 ± 28 IL-10 (OD): 0.26 ± 0.04 **Other Biomarkers:** Proliferation of peripheral lymphocytes (SI): 3.7 ± 0.8	**Pro-Inflammatory:** IL-2 (pg/mL): 760 ± 275 INF-γ (OD): 0.17 ± 0.05 **Anti-Inflammatory:** IL-4 (pg/mL): 145 ± 34 IL-10 (OD): 0.17 ± 0.03 **Other Biomarkers:** Proliferation of peripheral lymphocytes (SI): 3.9 ± 0.7
Molaie, 2012 [24] **Participants:** High background radiation vs. low background residential radiation	**Pro-Inflammatory:** IL-2 (μg): 159.2 (134.8–233.8) **Anti-Inflammatory:** IL-4 (μg): 225.6 (151.9–370.7) **Other Biomarkers:** NBT complete (%): 50 (21–84) NBT incomplete (%): 20 (10–50) NBT zero (%): 10 (0.0–20) Antioxidant levels (μmol): 873 (766–1032) Chemotaxis (μm): 115 (94–138)	**Pro-Inflammatory:** IL-2 (μg): 155.45 (0.873–116.29) **Anti-Inflammatory:** IL-4 (μg): 45.05 (26.82–61.88) **Other Biomarkers:** NBT complete (%): 99 (95–99) NBT incomplete (%): 0 (0–0) NBT zero (%): 1 (1–5) Antioxidant levels (μmol): 967 (870–1129) Chemotaxis (μm): 123 (97–152)
Leng, 2015 [25] **Participants:** Uranium miner cohort	**Pro-Inflammatory:** **In lymphoblastic cell lines** GG homozygous genotype IL-6: 6.18 ± 0.33 AG heterozygous genotype IL-6: 6.19 ± 0.33 AA genotype IL-6: 6.36 ± 0.37 **In human bronchial epithelial cells:** GG homozygous genotype IL-6: 0.0016 ± 0.0019 AG heterozygous genotype IL-6: 0.0031 ± 0.0036 AA genotype IL-6: 0.0041 ± 0.0038	
Li, 2018 [26] **Participants:** Offspring cohort and third-generation cohort residential radiation	Offspring cohort, Cycle 7: **Pro-Inflammatory:** MPO, ng/mL: 40.7 ± 22.6 8-epi-PGF2α, ng/mmol creatinine: 111 ± 63.6 CRP, mg/L: 2.27 ± 2.53 ICAM-1, ng/mL: 245 ± 58.0 MCP-1, pg/mL: 312 ± 104 P-selectin, ng/mL: 35.2 ± 13.3 TNF-α, pg/mL: 1.28 ± 0.639 TNFR-2, pg/mL: 2082 ± 664 **Other Biomarkers:** Fibrinogen, mg/dL: 372 ± 71.9 Third-generation cohort, Cycle 1: **Pro-Inflammatory:** CRP, mg/L: 1.11 ± 1.38 ICAM-1, ng/mL: 240 ± 57.8 IL-6, pg/mL: 1.37 ± 0.909 MCP-1, pg/mL: 331 ± 110 P-selectin, ng/mL: 45.4 ± 16.8 TNFR-2, pg/mL: 2180 ± 524 **Other Biomarkers:** Fibrinogen, mg/dL: 331 ± 66.1	
Blomberg, 2020 [27] **Participants:** Exposed to residential radon	**Pro-Inflammatory:** CRP (mg/L): first visit—1.6 (0.76–3.0); all visits—1.4 (0.71–2.7) ICAM -1 (ng/dL): first visit—288 (247–332); all visits—275 (237–323) VCAM-1 (ng/dL): first visit—987 (811–1210); all visits—978 (792–1220) **Other Biomarkers:** Fibrinogen (mg/dL): first visit—332 (296–378); all visits—328 (285–376)	
Autsavapromporn, 2021 [28] **Participants:** Lung cancer and participants without lung cancer: HRR (exposed) and LRR group (not exposed)	Lung cancer: **Pro-Inflammatory:** Il-8: 5.38 ± 4.01 MIF: 168.55 ± 343.23 TNF- α: 1.73 ± 0.68 VEGF: 56.47 ± 54.47 **Other Biomarkers:** Cyfra 21-1: 2464.49 ± 3454.32 CEA: 1581.21 ± 2084.37 HE4: 704.49 ± 898.70 HRR group: **Pro-Inflammatory:** Il-8: 3.61 ± 1.98 MIF: 148.68 ± 161.27 TNF- α: 1.63 ± 0.48 VEGF: 26.32 ± 21.67 **Other Biomarkers:** Cyfra 21-1: 332.91 ± 245.92 CEA: 355.77 ± 267.27 HE4: 545.26 ± 373.61	LRR: **Pro-Inflammatory:** Il-8: 1.92 ± 1.76 MIF: 98.89 ± 119.56 TNF- α: 1.61 ± 0.44 VEGF: 24.66 ± 14.46 **Other Biomarkers:** Cyfra 21-1: 219.58 ± 110.24 CEA: 240.20 ± 119.48 HE4: 540.77 ± 423.90
Purnami, 2023 [29] **Participants:** Exposed and not exposed residential radon	**Pro-Inflammatory:** TNF-α pg/mg: 3.99 (1.5–10.06) IFN-γ pg/mg: 2.31 (0.90–6.70) **Anti-Inflammatory:** IL-4 pg/mg: 4.71 (2.25–15.60) IL-10 pg/mg: 0.73 (0.17–1.77)	**Pro-Inflammatory:** TNF-α pg/mg: 4.85 (2.00–9.13) IFN-γ pg/mg: 2.75 (1.10–6.31) **Anti-Inflammatory:** IL-4 pg/mg: 5.43 (3.63–11.57) IL-10 pg/mg: 1.05 (0.16–9.18)
Zhang, 2023 [30] **Participants:** Exposed and not exposed to residential radon stratified by the smoking status	**Pro-Inflammatory:** CRP, mg/L: never smokers Zone 1 + Zone 2—3.2 ± 5.7; CRP, mg/L: ever smokers Zone 1 + Zone 2—3.8 ± 5.3	**Pro-Inflammatory:** CRP, mg/L: never smokers Zone 3—4.3 ± 7.4; CRP, mg/L: ever smokers Zone 3—4.9 ± 9.7
**Bronchoalveolar lavage fluid**		
Popp, 2000 [31] **Participants:** Study group (URAN) and healthy control group (CON); patients with asbestosis or fibrosis (FIBR); newly diagnosed lung cancer (CANC)	URAN: **Pro-Inflammatory:** TNF-α (units/mL BALF): 4.7 ± 3.3 **Other Biomarkers:** Fibrinogen (ng/mL BALF): 260.1 ± 186.6 Sum of surfactant factors (μg/mL BALF): 11.1 ± 5.2 Macrophages (per 1000 macrophages): 48.7 ± 15.1 Micronuclei in macrophages with two nuclei (per 1000 macrophages): 25.6 ± 34.1 Cell number (cells/mL BALF): 61.6 ± 69.0	FIBR: **Pro-Inflammatory:** TNF-α (units/mL BALF): 3.8 ± 2.4 **Other Biomarkers:** Fibrinogen (ng/mL BALF): 200.4 ± 198.5 Sum of surfactant factors (μg/mL BALF): 12.8 ± 9.5 Macrophages (per 1000 macrophages): 51.5 ± 18.9 Micronuclei in macrophages with two nuclei (per 1000 macrophages): 17.2 ± 20.7 Cell number (cells/mL BALF): 104.5 ± 132.8 CANC: **Pro-Inflammatory:** TNF-α (units/mL BALF): 3.1 ± 2.4 **Other Biomarkers:** Fibrinogen (ng/mL BALF): 246.6 ± 318.2 Sum of surfactant factors (μg/mL BALF): 10.4 ± 7.2 Macrophages (per 1000 macrophages): 55.8 ± 19.5 Micronuclei in macrophages with two nuclei (per 1000 macrophages): 18.3 ± 32.0 Cell number (cells/mL BALF): 125.5 ± 132.6 CON: **Other Biomarkers:** Macrophages (per 1000 macrophages): 47.9 ± 12.8 Micronuclei in macrophages with two nuclei (per 1000 macrophages): 9.9 ± 15.0
**Saliva**		
Taylor, 2022 [33] **Participants:** Children living in high radon concentration area	**Pro-Inflammatory:** CRP pg/mL: 319.74 ± 1671.54 IL-1 β pg/mL: 115.0 ± 134.32 IL-6 pg/mL: 7.21 ± 10.23 IL-8: 881.89 ± 675.63 TNF-α: 5.10 ± 5.18	

Abbreviations: 8-epi-PGF2α—8-epi-prostaglandin F2 alpha; CRP—C-reactive protein; ICAM-1—intercellular adhesion molecule 1; IL-1β—interleukin 1 beta; IL-2—interleukin 2; IL-4—interleukin 4; IL-6—interleukin 6; IL-8—interleukin 8; IL-10—interleukin 10; INF-γ—interferon gamma; MCP-1—monocyte chemoattractant protein 1; MIF—macrophage migration inhibitory factor; MPO—myeloperoxidase; P-selectin—platelet selectin; SD—standard deviation; TNF-α—tumor necrosis factor alpha; TNFR-2—tumor necrosis factor receptor 2; VCAM-1—vascular cell adhesion molecule 1; VEGF—vascular endothelial growth factor.

**Table 3:** Newcastle–Ottawa risk of bias (quality) assessment results.

Study	Selection	Comparability	Exposure	Total
Case–control studies				
Popp, 2000 [31]	3	1	3	7
Attar, 2007 [20]	4	1	3	8
Molaie, 2012 [24]	3	1	3	7
Leng, 2015 [25]	4	1	3	8
Autsavapromporn, 2021 [28]	4	1	2	7
Purnami, 2023 [29]	4	1	3	8
Cross-sectional studies				
Li, 2018 [26]	3	1	2	6
Blomberg, 2020 [27]	3	1	2	6
Zhang, 2023 [30]	3	1	2	6
Taylor, 2022 [33]	3	1	2	6

**Table 4:** Evaluation of evidence certainty using the GRADE framework.

Outcome	Study Design	Risk of Bias	Inconsistency	Indirectness	Imprecision	Publication Bias	Certainty of Evidence
Pooled mean CRP level	Meta-analysis of observational studies	Low	Serious	Not serious	Not serious	Possible	Low
Pooled mean TNF-α level	Meta-analysis of observational studies	Low	Serious	Not serious	Not serious	Possible	Low

Abbreviations: CRP—C-reactive protein; TNF-α—tumor necrosis factor alpha.

## Data Availability

The original contributions presented in this study are included in this article. Further inquiries can be directed to the corresponding author.

## Abbreviations

The following abbreviations are used in this manuscript:

8-epi-PGF2α	8-epi-prostaglandin F2 alpha
CANC	Patients with lung cancer
CON	Healthy controls
CRP	C-reactive protein
CRR	Chronic residential radon
EPA	Environmental Protection Agency
FIBR	Patients with lung asbestosis or fibrosis of the lung
HRR	High residential radon
ICAM-1	Intercellular adhesion molecule 1
ICRP	International Commission on Radiological Protection
IL-1β	Interleukin 1 beta
IL-2	Interleukin 2
IL-4	Interleukin 4
IL-6	Interleukin 6
IL-8	Interleukin 8
IL-10	Interleukin 10
INF-γ	Interferon gamma
LRR	Low residential radon
MCP-1	Monocyte chemoattractant protein 1
MIF	Macrophage migration inhibitory factor
MPO	Myeloperoxidase
NAS	Normative Aging Study
P-selectin	Platelet selectin
PRISMA	Preferred Reporting Items for Systematic Reviews and Meta-Analyses
SD	Standard deviation
TNF-α	Tumor necrosis factor alpha
TNFR-2	Tumor necrosis factor receptor 2
URAN	Uranium miners
VCAM-1	Vascular cell adhesion molecule 1
VEGF	Vascular endothelial growth factor
WLM	Working level month
